# Cine DENSE strain imaging of the right ventricle: improved methods and initial experience in heart failure

**DOI:** 10.1186/1532-429X-16-S1-P4

**Published:** 2014-01-16

**Authors:** Sophia Cui, Andrew D Gilliam, Kenneth C Bilchick, Frederick H Epstein

**Affiliations:** 1 Biomedical Engineering, University of Virginia, Charlottesville, Virginia, USA; 2 A.D. Gilliam Consulting, Providence, Rhode Island, USA; 3 Cardiovascular Medicine, University of Virginia, Charlottesville, Virginia, USA; 4 Radiology, University of Virginia, Charlottesville, Virginia, USA

## Background

**Figure 1:**
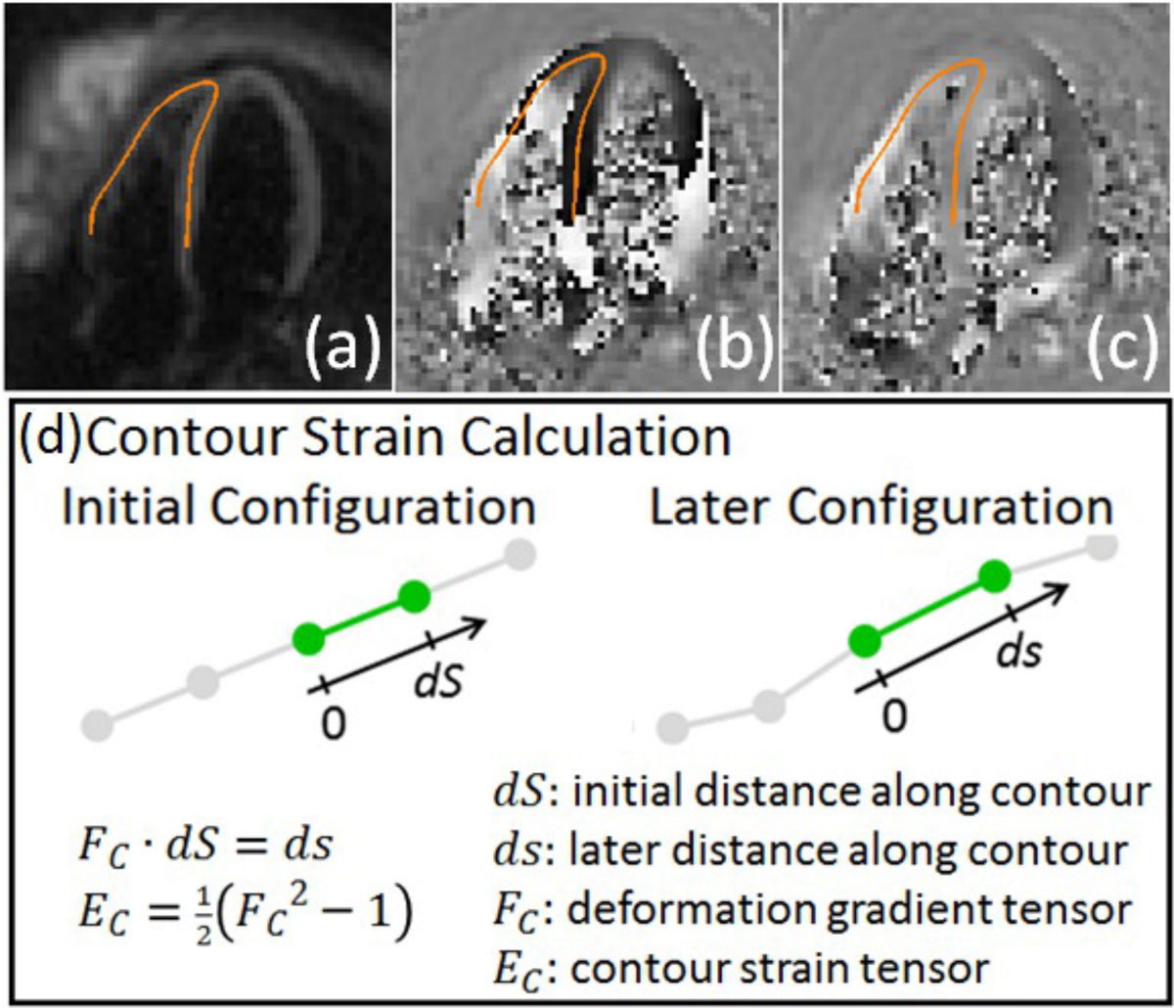
**A user defined RV contour indicated in orange shown on (a) magnitude-reconstructed and (b, c) phase-reconstructed DENSE images encoded for (b) horizontal displacement and (c) vertical displacement**. The Lagrangian strain calculated along the RV contour is described in (d).

Right ventricular (RV) dysfunction occurs in many cardiovascular conditions including pulmonary hypertension, congenital heart disease, and left-sided heart failure (HF), and its presence often alters patient management. Myocardial strain imaging has been used to assess left ventricular (LV) dysfunction; however, due to its thin wall, complex shape, and eccentric motion, strain analysis of the RV is more challenging and typically relies on manual delineation of tissue. Cine Displacement Encoding with Stimulated Echoes (DENSE) provides adequate image quality and spatial resolution to quantify RV function (Figure 1a, b, c), and the present challenge lies in developing more automated analysis methods.

## Methods

Building upon prior methods for LV analysis [1,2], we developed improved algorithms for semi-automatically analyzing RV strain. The first stage of RV analysis is anatomical delineation, locating DENSE observations attributable to underlying RV tissue. The algorithm requires user definition of RV anatomy on a single long-axis frame using a line contour (Figure 1a, b, c). Next, using a phase-quality-guided path-finding algorithm for phase unwrapping and using spatial interpolation with radial basis functions (RBF), the user-defined contour is propagated to all other cardiac phases through a process termed motion guided segmentation (MGS) [1]. With MGS, we project the manually defined RV contour to other cardiac phases using RBF interpolation of the displacement field. Lastly, material point trajectories are fit through time via a high order polynomial function. The final phase of RV analysis is strain estimation. Given the thin structure of the RV, we used a 1D contour strain, as illustrated in Figure 1. These new algorithms were applied to long-axis cine DENSE images acquired from 18 subjects without heart disease and 16 subjects with heart failure. Four data sets from HF patients were excluded due to aliasing artifacts. The RV was divided into two segments, namely the RV free wall and the septum.

## Results

**Figure 2:**
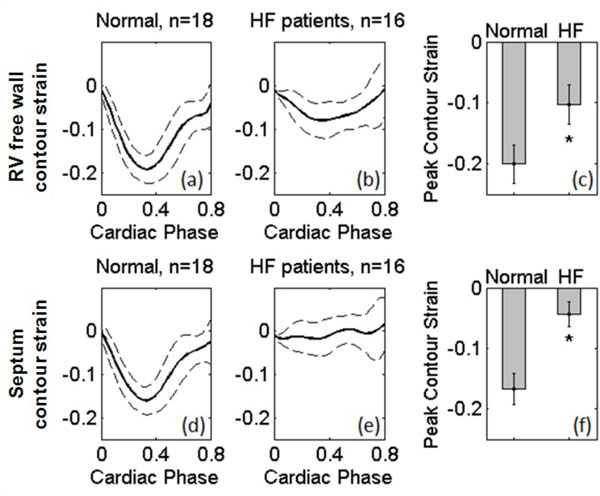
**Mean RV contour strain for 18 subjects without heart disease (a. d) and 16 subjects with heart failure (b, e)**. Segmental data are shown for the RV free wall (a, b) and the septum (d, e). (c, f) RV peak contour strain for (c) the RV free wall and (f) the septum demonstrate dysfunction in heart failure patients compared to subjects without heart disease (*p < 0.01). All data shown are mean ± standard deviation.

For the 34 datasets that were analyzed, no manual editing of the automatically-propagated RV contours was needed. For the 18 subjects without heart disease, the mean contour strain (Figure 2a, d) for the RV free wall and the septum had peak values of -0.20 ± 0.03 and -0.17 ± 0.03, respectively, whereas these values were -0.10 ± 0.03 and -0.04 ± 0.02, respectively, (p < 0.01 vs. without heart disease) for the 16 HF subjects.

## Conclusions

The new DENSE analysis methods successfully propagated RV contours to sequential cardiac frames and computed RV contour strains. Using these methods, cine DENSE detected abnormal RV strain in HF patients compared to subjects without heart disease.

## Funding

NIH K23 grant HL094761 and American Heart Association Grant-in-Aid 12GRNT12050301.
